# Supplementary material for: Localized wastewater surveillance showed correlation but no early warning during Bengaluru’s Omicron wave
Source: PLOS Glob Public Health. 2026 Apr 10;6(4):e0004684. doi: 10.1371/journal.pgph.0004684 (PMC13068238; doi:10.1371/journal.pgph.0004684)
Supplement: S1 Fig — (PDF) [file pgph.0004684.s001.pdf]

S1 Fig. Drainage pipes from wards leading to the Hebbal STP

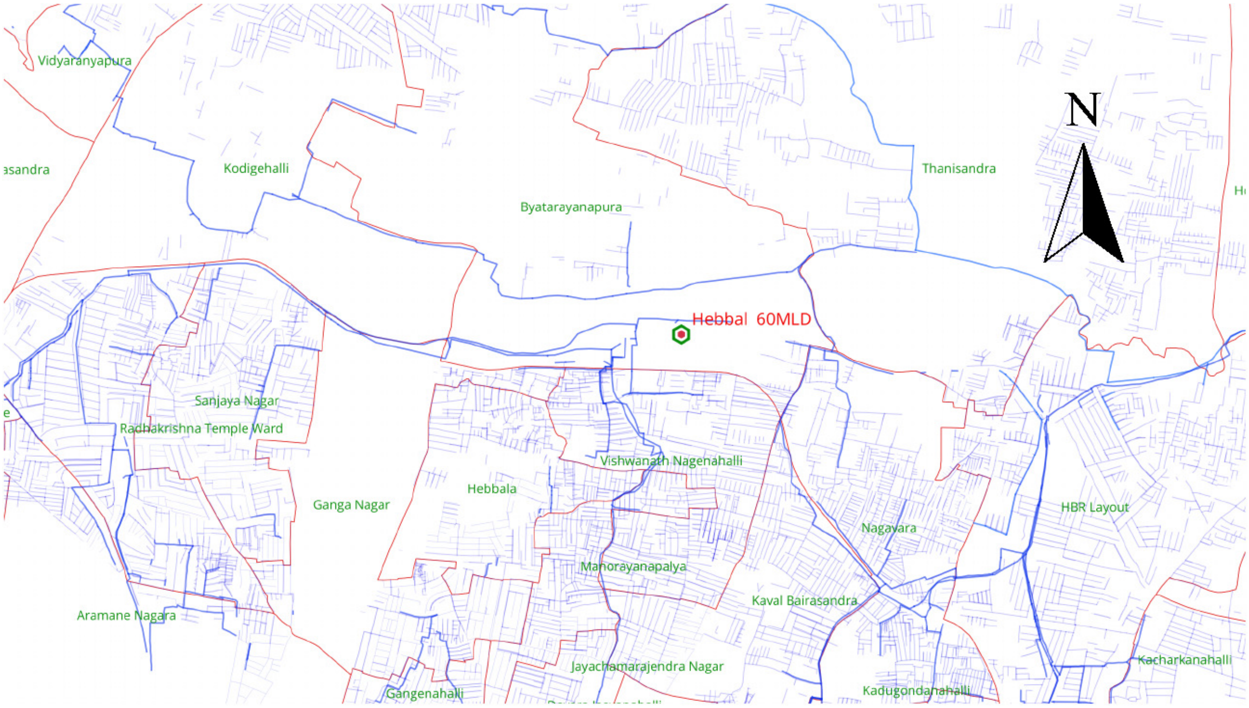

We see a large sewer line (thick blue line) running from Sanjay Nagar ward directly into the Hebbal STP. The smaller sewer lines from residential areas of Sanjay Nagar connect to the larger sewer line. After we get the flow direction from the terrain, we infer that Sanjay Nagar is connected to the Hebbal STP.

Source

Base layer: Bangalore BBMP wards
Sewer lines: BWSSB Sewerage Lines Maps of Bengaluru
License: Open Data Commons Open Database License (ODbL) v1.0.
