# Supplementary material for: Localized wastewater surveillance showed correlation but no early warning during Bengaluru’s Omicron wave
Source: PLOS Glob Public Health. 2026 Apr 10;6(4):e0004684. doi: 10.1371/journal.pgph.0004684 (PMC13068238; doi:10.1371/journal.pgph.0004684)
Supplement: S2 Fig — (PDF) [file pgph.0004684.s002.pdf]

S2 Fig. BWSSB sewer lines coverage

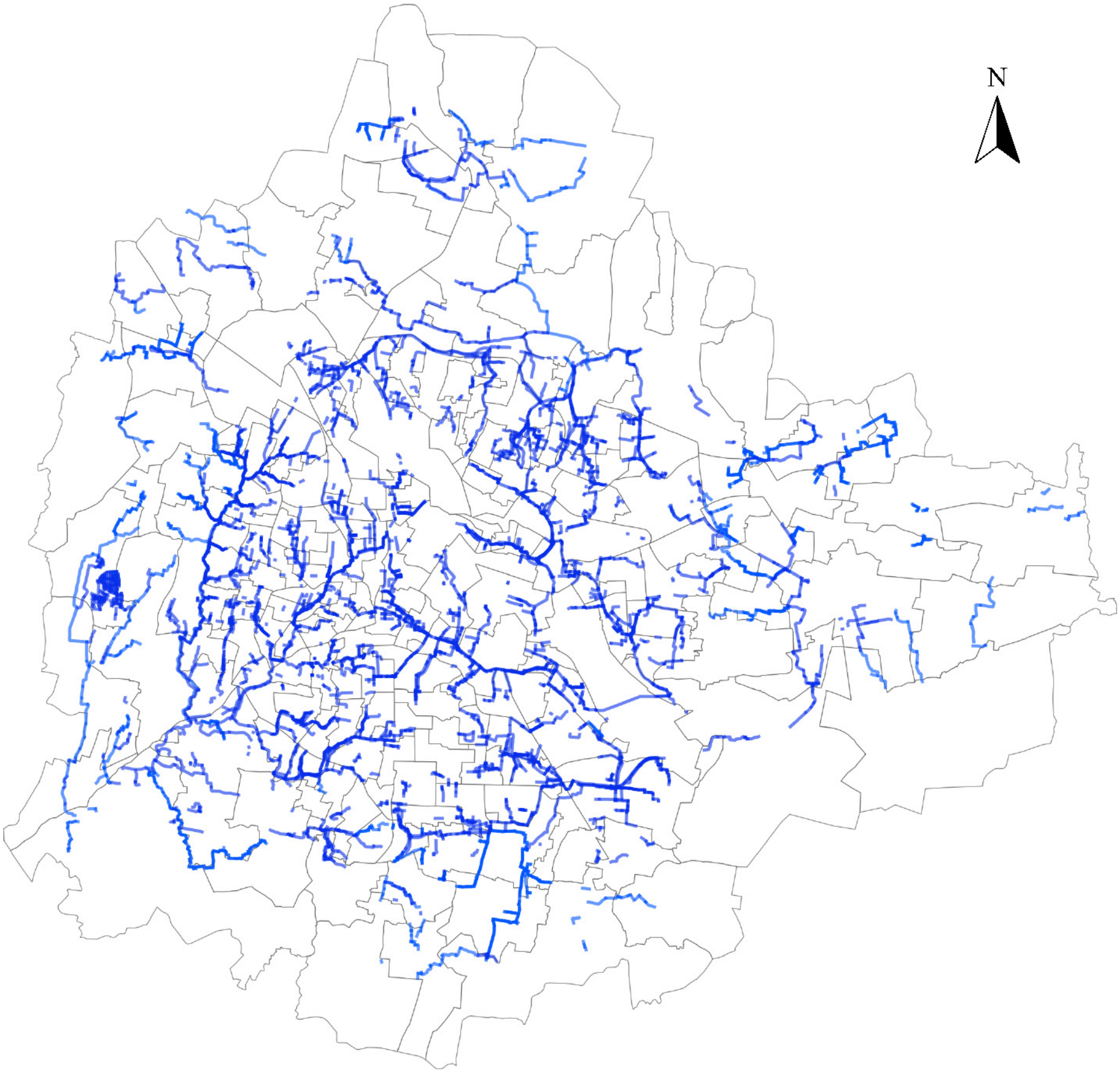

The diameter of the sewer pipes is greater than 300mm.

Source

Base layer: Bangalore BBMP wards
Sewer lines: BWSSB Sewerage Lines Maps of Bengaluru
License: Open Data Commons Open Database License (ODbL) v1.0.
