# Supplementary material for: Localized wastewater surveillance showed correlation but no early warning during Bengaluru’s Omicron wave
Source: PLOS Glob Public Health. 2026 Apr 10;6(4):e0004684. doi: 10.1371/journal.pgph.0004684 (PMC13068238; doi:10.1371/journal.pgph.0004684)
Supplement: S3 Fig — (PDF) [file pgph.0004684.s003.pdf]

S3 Fig. A scatter plot of viral loads and cases from four STPs

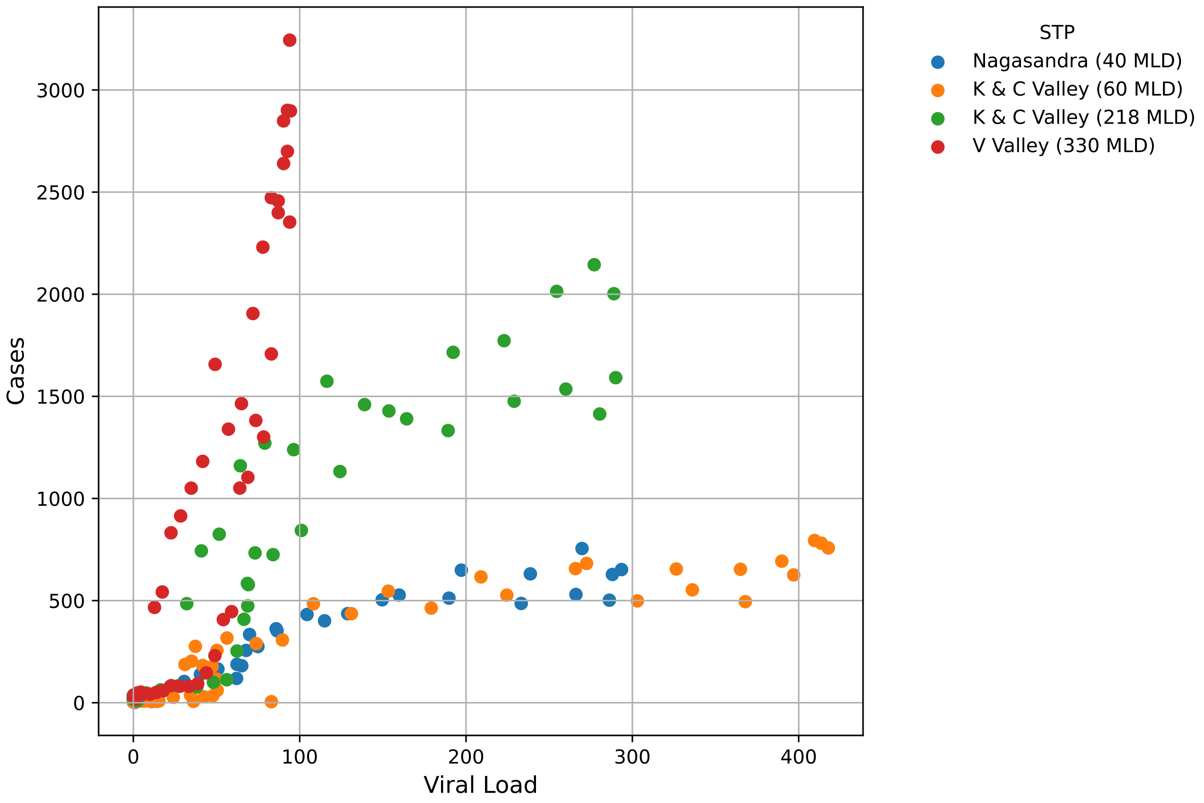

Data points from an STP roughly lie around a straight line. A similar trend holds for other STPs also.
