# Supplementary figures and images for: Localized wastewater surveillance showed correlation but no early warning during Bengaluru’s Omicron wave
Source: PLOS Glob Public Health. 2026 Apr 10;6(4):e0004684. doi: 10.1371/journal.pgph.0004684 (PMC13068238; doi:10.1371/journal.pgph.0004684)

**S4 Fig. Error bars for viral load estimates at STPs in California.**

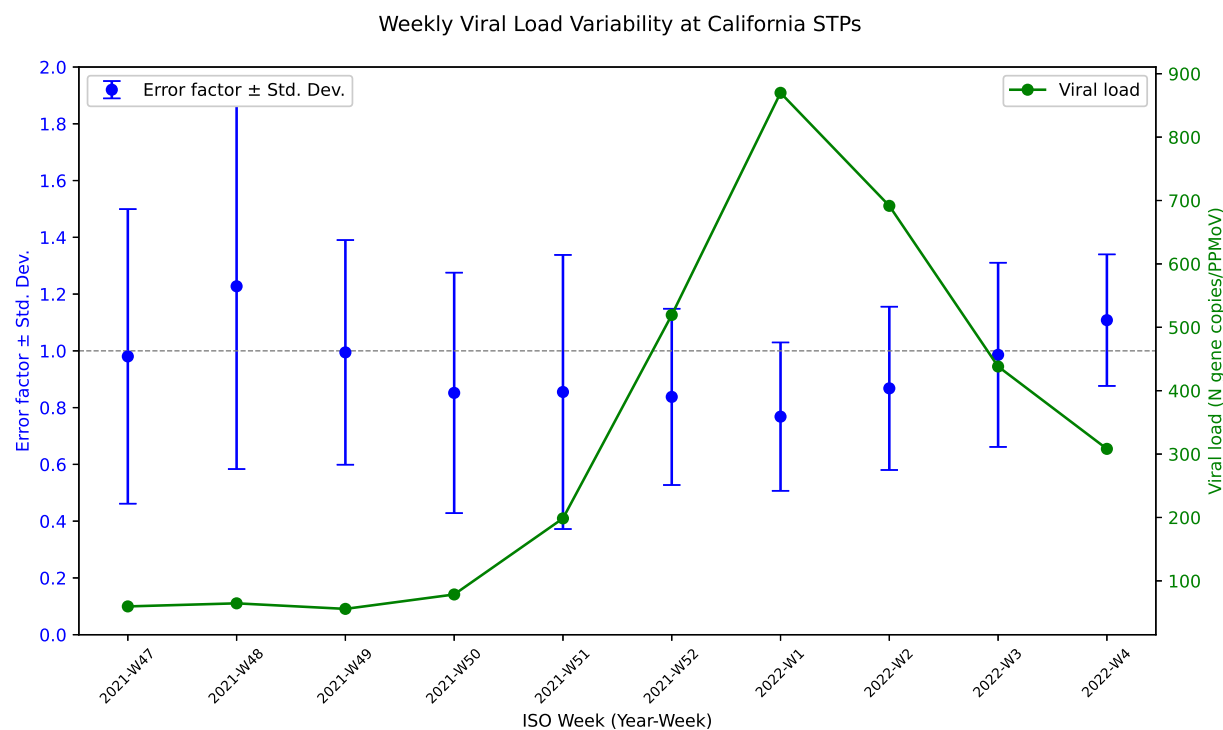

Supplement: S4 Fig — (PDF) [file pgph.0004684.s004.pdf]

**S5 Fig. Error bars for cases estimates at STP catchments in Bengaluru**

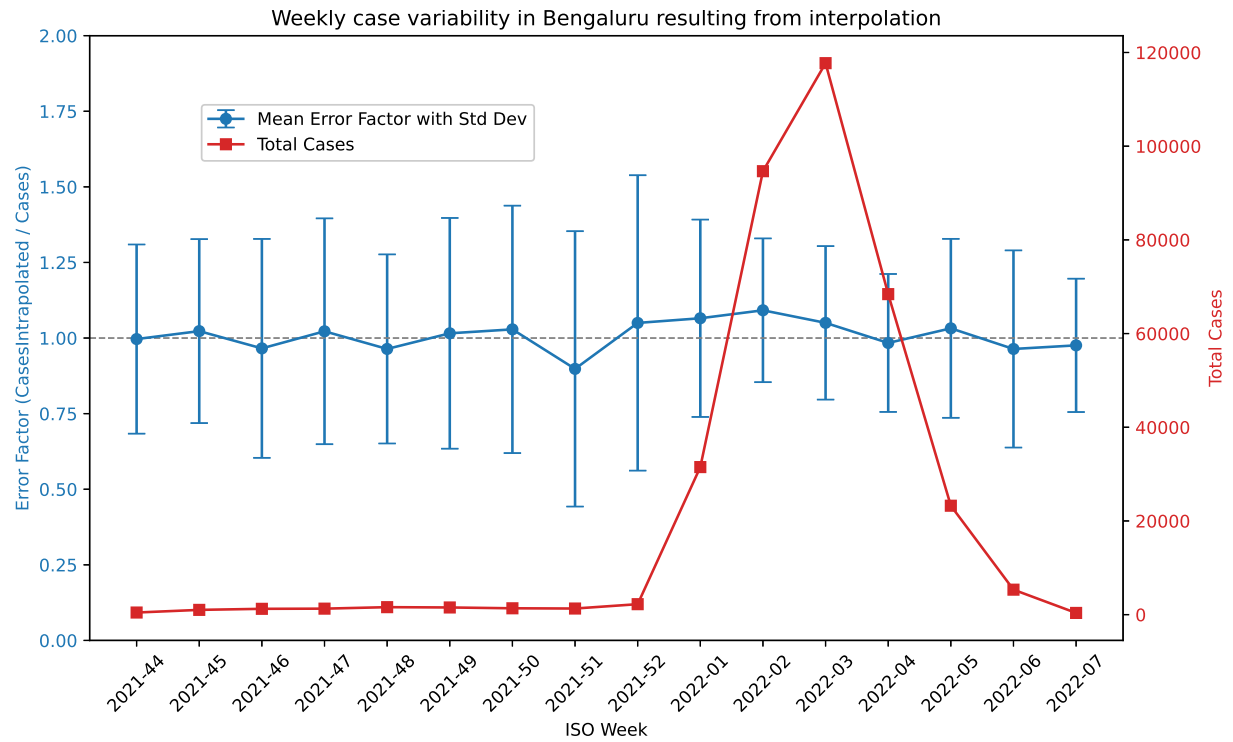

Supplement: S5 Fig — (PDF) [file pgph.0004684.s005.pdf]
