# Supplementary material for: Localized wastewater surveillance showed correlation but no early warning during Bengaluru’s Omicron wave
Source: PLOS Glob Public Health. 2026 Apr 10;6(4):e0004684. doi: 10.1371/journal.pgph.0004684 (PMC13068238; doi:10.1371/journal.pgph.0004684)
Supplement: S6 Fig — (PDF) [file pgph.0004684.s006.pdf]

S6 Fig. Distribution of correlation values between a vector and its noisy version

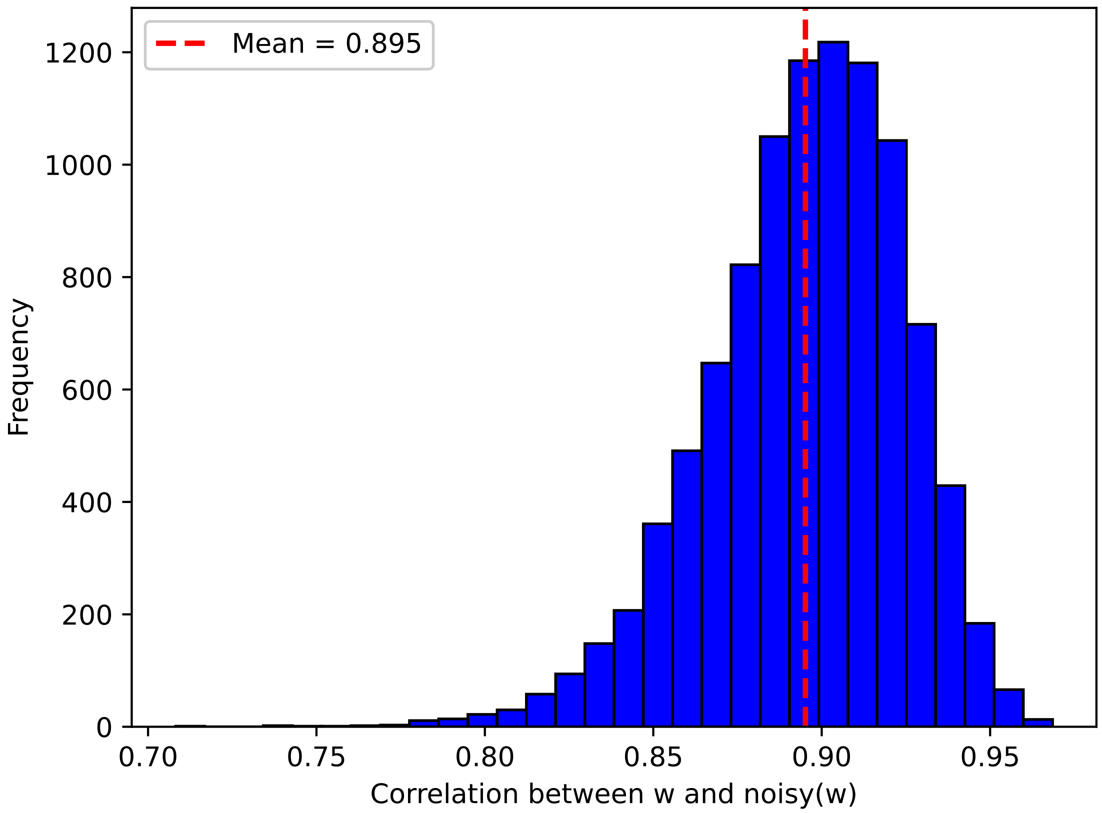

The data is from 10000 trials. In a trial, a random unit vector $\mathbf{w}$ is picked, and each value in $\mathbf{w}$ is perturbed with 25% Gaussian noise. The histogram shows the frequency of correlations between $\mathbf{w}$ and the $\mathbf{w}$ with noise.
