# Supplementary material for: Localized wastewater surveillance showed correlation but no early warning during Bengaluru’s Omicron wave
Source: PLOS Glob Public Health. 2026 Apr 10;6(4):e0004684. doi: 10.1371/journal.pgph.0004684 (PMC13068238; doi:10.1371/journal.pgph.0004684)
Supplement: S1 Table — (PDF) [file pgph.0004684.s007.pdf]

S1 Table. Parameter values of the pre-and-post distributions of alternate slopes.

STP name	Viral load rate		Case rate	
	$f_0 : \mathcal{N}(\mu_0, \sigma_0)$	$f_1 : \mathcal{N}(\mu_1, \sigma_1)$	$f_0 : \mathcal{N}(\mu_0, \sigma_0)$	$f_1 : \mathcal{N}(\mu_1, \sigma_1)$
Agaram (35 MLD)	(0.47, 1.18)	(28.40, 43.50)	(-0.13, 4.33)	(47.93, 84.82)
Bellandur (90 MLD)	(-0.03, 0.05)	(18.77, 33.03)	(1.36, 2.71)	(30.41, 61.95)
Chikkabanavara (5 MLD)	(0.22, 0.45)	(18.36, 20.33)	(-0.14, 0.42)	(5.50, 16.71)
Chikkabegur (5 MLD)	(-0.02, 0.23)	(11.53, 12.12)	(-0.62, 2.16)	(10.71, 28.17)
Cubbon Park (4 MLD)	(-0.00, 0.09)	(1.97, 4.87)	(0.07, 0.56)	(1.98, 12.30)
Doddabele (60 MLD)	(0.01, 0.66)	(19.21, 33.53)	(0.28, 2.45)	(24.59, 90.17)
Halasuru (2 MLD)	(0.01, 0.08)	(35.68, 57.74)	(-0.12, 0.26)	(3.17, 4.04)
Hebbal (100 MLD)	(0.18, 0.61)	(5.42, 14.44)	(0.41, 3.31)	(40.85, 81.77)
Hulimavu (10 MLD)	(-0.00, 0.23)	(8.75, 9.54)	(0.46, 1.90)	(12.21, 39.06)
Jakkur (15 MLD)	(0.13, 0.86)	(6.91, 25.35)	(0.33, 1.94)	(6.14, 17.27)
K & C Valley (218 MLD)	(-0.02, 0.27)	(11.32, 12.25)	(-0.90, 4.17)	(41.64, 95.55)
K & C Valley (60 MLD)	(-2.41, 10.90)	(14.49, 21.53)	(0.14, 2.09)	(26.86, 60.57)
K R Puram Old (20 MLD)	(-0.12, 0.46)	(11.05, 14.17)	(-0.55, 2.09)	(14.97, 27.79)
Kadabeesanahalli (50 MLD)	(-0.03, 0.26)	(7.61, 10.74)	(0.56, 5.21)	(17.15, 36.17)
Kadugodi (6 MLD)	(-0.04, 1.09)	(14.29, 19.64)	(-0.30, 2.57)	(7.86, 28.39)
Kempambudhi (1 MLD)	(0.05, 0.59)	(15.00, 26.15)	(-0.09, 1.02)	(0.07, 12.26)
Kengeri (60 MLD)	(0.20, 0.85)	(1.40, 7.16)	(-0.37, 1.98)	(5.68, 43.60)
Lalbagh (1.5 MLD)	(0.15, 0.40)	(12.91, 24.57)	(-0.19, 0.97)	(-0.26, 14.23)
Mailasandra (75 MLD)	(1.35, 2.80)	(8.07, 8.49)	(-0.91, 3.96)	(5.05, 76.98)
Mallathahalli (5 MLD)	(-0.06, 0.25)	(4.07, 15.39)	(-0.01, 0.34)	(4.40, 8.32)
Nagasandra (40 MLD)	(0.03, 0.13)	(10.80, 14.98)	(0.05, 1.71)	(27.62, 55.83)
Rajacanal (80 MLD) & Horamavu (20 MLD)	(0.22, 0.70)	(23.46, 34.00)	(0.69, 3.70)	(17.62, 113.88)
Sarakki (5 MLD)	(-0.02, 0.10)	(28.52, 55.83)	(0.03, 1.88)	(22.01, 34.86)
V Valley (330 MLD)	(0.02, 0.25)	(3.86, 1.58)	(-0.43, 4.40)	(60.00, 241.43)
Yelahanka (10 MLD)	(-0.14, 0.25)	(10.75, 37.17)	(-0.11, 0.48)	(-1.00, 28.61)
Yellamallappachetty (15 MLD)	(-0.12, 0.29)	(0.86, 2.53)	(-0.55, 2.82)	(5.64, 22.87)
Bengaluru	(0.04, 0.47)	(10.69, 10.28)	(4.67, 14.73)	(876.14, 789.42)

† For Lalbagh and Yelahanka, the post-change case rate is negative due to a sudden drop in cases on a particular day.
