# Supplementary material for: Localized wastewater surveillance showed correlation but no early warning during Bengaluru’s Omicron wave
Source: PLOS Glob Public Health. 2026 Apr 10;6(4):e0004684. doi: 10.1371/journal.pgph.0004684 (PMC13068238; doi:10.1371/journal.pgph.0004684)
Supplement: S2 Table — (PDF) [file pgph.0004684.s008.pdf]

S2 Table. Correlation between daily viral loads and values interpolated from weekly samples in California.

STP Name	# Measurements	Correlation $\rho_{\nu\tilde{\nu}}$
Sacramento Regional Wastewater Treatment Plant	78	0.91
City of Sunnyvale Water Pollution Control Plant	78	0.82
San Jose-Santa Clara Regional Wastewater Facility	78	0.80
Oceanside Water Pollution Control Plant	72	0.85
Palo Alto Regional Water Quality Control Plant	77	0.71
Silicon Valley Clean Water	78	0.70
