# Supplementary material for: Localized wastewater surveillance showed correlation but no early warning during Bengaluru’s Omicron wave
Source: PLOS Glob Public Health. 2026 Apr 10;6(4):e0004684. doi: 10.1371/journal.pgph.0004684 (PMC13068238; doi:10.1371/journal.pgph.0004684)
Supplement: S3 Table — (PDF) [file pgph.0004684.s009.pdf]

S3 Table. Correlation between daily cases and interpolated cases from weekly samples in Bengaluru.

STP Name	Correlation $\rho_{\mathbf{c}\tilde{\mathbf{c}}}$	# Samples
Agaram (35 MLD)	0.98	9
Bellandur (90 MLD)	0.99	9
Chikkabanavara (5 MLD)	0.95	10
Chikkabegur (5 MLD)	0.98	9
Cubbon Park (4 MLD)	0.99	9
Doddabele (60 MLD)	0.99	8
Halasuru (2 MLD)	0.97	9
Hebbal (100 MLD)	0.99	8
Hulimavu (10 MLD)	0.97	9
Jakkur (15 MLD)	0.97	11
K & C Valley (218 MLD)	0.99	10
K & C Valley (60 MLD)	0.99	10
K R Puram Old (20 MLD)	0.98	9
Kadabeesanahalli (50 MLD)	0.99	10
Kadugodi (6 MLD)	0.94	8
Kempambudhi (1 MLD)	0.95	8
Kengeri (60 MLD)	0.99	7
Lalbagh (1.5 MLD)	0.96	9
Mailasandra (75 MLD)	0.97	8
Mallathahalli (5 MLD)	0.94	9
Nagasandra (40 MLD)	0.98	10
Rajacanal (80 MLD) and Horamavu (20 MLD)	0.99	19
Sarakki (5 MLD)	0.99	9
V Valley (330 MLD)	0.98	7
Yelahanka (10 MLD)	0.92	12
Yellamallappachetty (15 MLD)	0.99	9
