# Supplementary material for: Localized wastewater surveillance showed correlation but no early warning during Bengaluru’s Omicron wave
Source: PLOS Glob Public Health. 2026 Apr 10;6(4):e0004684. doi: 10.1371/journal.pgph.0004684 (PMC13068238; doi:10.1371/journal.pgph.0004684)
Supplement: S4 Table — (PDF) [file pgph.0004684.s010.pdf]

Description of supplementary data files.

Folder	File	Description
mapfiles	BLRWardsSTPs.qgz	A QGIS project file describing the ward boundaries, STP catchment areas and STP locations.
	BLRWardsSTPs.gpkg	A geopackage that contains all the information that feeds into BLR-WardsSTPs.qgz.
	SewerLines.qgz	A QGIS project describing the BWSSB sewer network of Bengaluru.
	SewerLines.gpkg	A geopackage that contains all the information that feeds into Sewer-Lines.qgz.
raw_data	DailyCases.csv	Raw daily reported cases in BBMP wards.
	ViralLoads.csv	Raw viral loads recorded at BWSSB STPs. (It may be noted that there is an inadvertent data-conversion error in [13, Supplementary Table S4]. As confirmed by the corresponding author of [13], the correct values of the raw viral loads (copies/ml) for December 2021 are 1.25x of those reported in column G of [13, Supplementary Table S4]. The correct values are available in ViralLoads.csv file.)
	ViralLoads_CT.xlsx	Raw viral loads with Ct values for three genes. Raw viral loads are the average of the viral loads for the three genes.
	StdWard.csv	Standardized names of all the wards in the raw cases data.
	StdSTP.csv	Standardized names of all the STPs in the raw viral load data.
	STPMap.csv	Mapping of STPs to composite STPs, if any.
	WardSTPMap.csv	The mapping of wards to STPs.
	SCAN*.csv	Viral loads from California STPs before 2022. Source: Boehm AB et. al. (2023) doi: 10.25740/cx529np1130
	wwscan*.csv	Viral loads from California STPs since 2022. Source: Boehm AB et. al. (2024) doi: 10.25740/hj801ns5929
data	stpviralcases.csv	Interpolated daily data of cases and viral loads corresponding to each STP. This is an input file for both the CUSUM algorithm and correlations.
	cityviralcases.csv	Aggregated daily data of cases and viral loads corresponding to Bengaluru city. This is an input file for both the CUSUM algorithm and correlations.
	california.csv	Viral loads at STPs in California combining 2021 and 2022 data.
code	PreprocessData.py	The Python file that preprocesses the files present within the raw_data folder and generates stpviralcases.csv and cityviralcases.csv.
	cusum.py	Implementation of the CUSUM algorithm in Python. The file takes either stpviralcases.csv or cityviralcases.csv as input and outputs the change points of cases and viral loads for threshold h=20 (Table 3, Table S1, Fig. 4, and all subplots of Fig. 5).
	cusum threshold.py	Outputs mean and standard deviations of lead times (Table 4).
	stp_confidence_intervals.py	Script that finds correlations between cases and viral loads with CIs (Table 2).
	california_validation.py	Outputs the correlation table for California STPs (Table S2).
	blr_cases_validation.py	Outputs the correlation table for Bengaluru cases (Table S3).
	california_error_bar.py	Script to plot weekly variability of viral loads (Fig. S4).
	blr_cases_error_bar.py	Script to plot error bars for weekly cases in Bengaluru (Fig. S5).
	stp_scatterplot.py	Outputs scatter plot of cases and viral loads (Fig. S3).
	corr_noise_25.py	Simulation to compute the distribution of correlation values between a vector and its noisy version (Fig. S6).
results		Folder where outputs of the above scripts are written.
