# Supplementary material for: Localized wastewater surveillance showed correlation but no early warning during Bengaluru’s Omicron wave
Source: PLOS Glob Public Health. 2026 Apr 10;6(4):e0004684. doi: 10.1371/journal.pgph.0004684 (PMC13068238; doi:10.1371/journal.pgph.0004684)
Supplement: S1 Text — (PDF) [file pgph.0004684.s011.pdf]

S1 Text. An example of computing weighted viral load

We illustrate the computation of weighted viral load via a toy example. Suppose STP A has a treatment capacity, C_A, of 50 million liters per day (MLD) and a daily viral load, V_A, of 2.5×10^3 copies/ml, while STP B has a treatment capacity, C_B, of 100 MLD and a daily viral load, V_B, of 3.5×10^3 copies/ml. Then,

$$\begin{aligned}\text{Weighted Viral Load} &= \frac{(V_A \cdot C_A) + (V_B \cdot C_B)}{C_A + C_B} \\ &= \frac{(2.5 \times 10^3 \cdot 50) + (3.5 \times 10^3 \cdot 100)}{50 + 100} \\ &= 3.17 \times 10^3 \text{ copies/ml.}\end{aligned}$$
