# Supplementary material for: Localized wastewater surveillance showed correlation but no early warning during Bengaluru’s Omicron wave
Source: PLOS Glob Public Health. 2026 Apr 10;6(4):e0004684. doi: 10.1371/journal.pgph.0004684 (PMC13068238; doi:10.1371/journal.pgph.0004684)
Supplement: S2 Text — (PDF) [file pgph.0004684.s012.pdf]

ಸಾರಾಂಶ

ಸಂಕ್ರಾಮಕ ರೋಗಗಳ ಹಬ್ಬು ಹರಡನ್ನು ಗಮನಿಸಲು ತ್ಯಾಜ್ಯ ನೀರಿನ ಮೇಲ್ವಿಚಾರಣೆ ಪರಿಣಾಮಕಾರಿಯಾದ ಸಾಧನವಾಗಿದೆ, SARS-CoV-2 ನಂತಹ ರೋಗಗಳನ್ನು ವಿಶ್ಲೇಷಿಸಲು. 2021ರ ಆಗಸ್ಟ್‌ನಲ್ಲಿ, ಟಾಟಾ ಇನ್ಸ್ಟಿಟ್ಯೂಟ್ ಫಾರ್ ಜೆನೆಟಿಕ್ಸ್ ಅಂಡ್ ಸೊಸೈಟಿ (TIGS) ನ ಸಂಶೋಧಕರ ತಂಡವು ಬೆಂಗಳೂರಿನಲ್ಲಿ ನಗರವ್ಯಾಪಿ ಮೇಲ್ವಿಚಾರಣಾ ಪ್ರಯತ್ನವನ್ನು ಪ್ರಾರಂಭಿಸಿತು, 28 ಮಲಮೂತ್ರ ಶುದ್ಧೀಕರಣ ಘಟಕಗಳಿಂದ (STPs) ವೈರಲ್ ಲೋಡ್ಗಳನ್ನು ವಿಶ್ಲೇಷಿಸಿತು. ಅವರ ಅಧ್ಯಯನವು ಒಟ್ಟಾರೆ ವೈರಲ್ ಲೋಡ್ಗಳು ಮತ್ತು ನಗರವ್ಯಾಪಿ COVID-19 ಪ್ರಕರಣಗಳ ಸಂಖ್ಯೆ ನಡುವಿನ ಬಲವಾದ ಸಂಬಂಧವನ್ನು ತೋರಿಸಿತು. ಆದರೆ, ಸ್ಥಳೀಯ ವೈದ್ಯಕೀಯ ಮಾಹಿತಿಯ ಕೊರತೆಯು STP ಮಟ್ಟದಲ್ಲಿ ಸೋಂಕಿನ ಪ್ರವರ್ತನೆಗಳನ್ನು ವಿಶ್ಲೇಷಿಸಲು ಸಾಧ್ಯವಾಗಲಿಲ್ಲ. ಈ ಮುಂದಿನ ಅಧ್ಯಯನದಲ್ಲಿ, ನಾವು ನಗರದ 198 ಆಡಳಿತಾತ್ಮಕ ಘಟಕಗಳಿಂದ ನಿಖರವಾದ ವೈದ್ಯಕೀಯ ಮಾಹಿತಿಯನ್ನು ಒಳಗೊಂಡಿದ್ದೇವೆ. ನಮ್ಮ ಅಧ್ಯಯನವು ವೈಯುಕ್ತಿಕ STP ನಲ್ಲಿ ವೈರಲ್ ಲೋಡ್ಗಳು ಮತ್ತು ಅದರ ಕ್ಯಾಚ್‌ಮೆಂಟ್ ಪ್ರದೇಶಗಳಲ್ಲಿ ಪ್ರಕರಣಗಳ ನಡುವೆ ಸಮಾನ ಪ್ರವರ್ತನೆಗಳನ್ನು ಕಂಡುಹಿಡಿಯುತ್ತದೆ. ನಮ್ಮ ಅಧ್ಯಯನವು ವೈರಲ್ ಲೋಡ್ಗಳು ಸ್ಥಳೀಯ ಪ್ರದೇಶಗಳಲ್ಲಿ ಪ್ರಕರಣದ ಮಾಹಿತಿಗಳೊಂದಿಗೆ ಹೊಂದಿಕೆಯಾಗುವುದನ್ನು ದೃಢಪಡಿಸುತ್ತದೆ. ಆದರೆ, ನಮ್ಮ ವಿಶ್ಲೇಷಣೆಯು ಮುಂಚಿನ ಸೂಚನೆ ಅಥವಾ ಮುಂಚೆಚ್ಚರಿಕೆಯ ಯಾವುದೇ ಗುರುತುಗಳನ್ನು ತೋರಿಸುವುದಿಲ್ಲ - ವೈರಲ್ ಲೋಡ್ಗಳು ಮತ್ತು ವರದಿಯಾದ ಪ್ರಕರಣಗಳು ಏಕಕಾಲದಲ್ಲಿ ಹೆಚ್ಚುತ್ತವೆ.ನಮ್ಮ ಅಧ್ಯಯನವು ಓಮಿಕ್ರಾನ್ ತರಂಗದ ಸಮಯಕ್ಕೆ ಮಾತ್ರ ಸೀಮಿತವಾಗಿದೆ.
