# Supplementary material for: Localized wastewater surveillance showed correlation but no early warning during Bengaluru’s Omicron wave
Source: PLOS Glob Public Health. 2026 Apr 10;6(4):e0004684. doi: 10.1371/journal.pgph.0004684 (PMC13068238; doi:10.1371/journal.pgph.0004684)
Supplement: Attachment — Submitted filename: PLOS_Rebuttal.pdf [file pgph.0004684.s013.pdf]

Responses to Reviewers' Comments for Manuscript PGPH-D-24-02064

Localized wastewater surveillance showed correlation but no early warning during Bengaluru's Omicron wave

Addressed Comments for Publication to

PLOS Global Public Health

by

Siva Athreya, Tarun Khandelwal, Jagadish Midthala, Chitra Pattabiraman,
Lakshminarayana Rao, Rajesh Sundaresan and Reshma Mohan Thattaramppilly

Dear Dr. Amarasiri,

Please find enclosed the revised version of our previous submission titled " Localized wastewater surveillance showed correlation but no early warning during Bengaluru's Omicron wave" with original manuscript number PGPH-D-24-02064. We want to thank you and the reviewers for the valuable comments. In this revision, we have addressed the queries raised during the review process. A summary of the main modifications and point-by-point responses to the comments are given below.

Sincerely,

Jagadish Midthala

Note: To enhance the legibility of this response letter, the editor's and reviewers' comments are typeset in boxes. Changes to the manuscript are given in boxes with gray background. Line numbers refer to the line numbers in the original manuscript. In the marked manuscript major changes are marked in blue color.

Authors' Response to the Editor

General Comments. The reviewers have raised several important questions related to the limited sample number and the generalization of the results.

Response:

We thank the reviewers for their careful reading of the manuscript and appreciate the detailed feedback given. We have revised our manuscript based on the reviewers' comments. The main changes are listed below.

1. We ran additional experiments to validate our method. We show that the number of samples is sufficient to establish a correlation between viral loads from wastewater and cases, during the Omicron wave.
2. We have added a new section on "Validating the sample size and interpolation method".
3. We have expanded the "Discussion" section.
4. As suggested, we have explicitly stated our claims and avoided broad generalizations.
5. Supporting data and code have been updated.

Authors' Response to Reviewer 1

General Comments. This manuscript presents a study analyzing correlations between SARS-CoV-2 viral loads in wastewater and COVID-19 case numbers in Bengaluru, India, during the Omicron wave. The authors examine data at both citywide and localized levels across 26 sewage treatment plants (STPs) from December 15, 2021, to January 15, 2022. The study addresses an important topic and offers some interesting insights, but there are several limitations that need to be addressed to strengthen the manuscript.

Response: Thank you for the comments. We have addressed the comments and strengthened the manuscript as suggested by the reviews. We provide a point by point response below and we hope that they are to the reviewer's satisfaction

Comment 1

One major concern is the limited number of viral load data points available. With weekly sampling over a one-month period, there are only 4-5 data points per STP. This small sample size may impact the reliability of the correlation analyses that are central to the study. The authors should consider providing a power analysis to assess if this sample size is sufficient for drawing robust conclusions. If not, the findings and claims may need to be qualified accordingly.

The authors should discuss the implications of having few data points, including the potential impact on the uncertainty of correlation estimates, sensitivity to outliers, and statistical power. Limitations related to sample size should be addressed in the discussion.

Response: Thank you for raising this important issue. We modified the methodology of correlation analysis to handle a larger time window (77 days). The number of samples per STP is now larger at about 8-9. We also have done away with the seven day smoothing of

cases. We have added a new Section titled "Validating the sample size and interpolation method". This section covers the following.

- (Interpolation Error): We have given viral load estimates of the interpolation method for California STPs (Fig. 6). Daily data was available for these STPs, but we deliberately subsample and interpolate, then compare the interpolation method's outcome with the true daily data. The approach gives us a way to estimate the standard error of the interpolation method, which is found to be about 25% during the period of the Omicron wave. We have also explained the sensitivity of the error bounds when the viral loads are close to zero, as it happens outside of the wave. We remove these outliers before calculating the error bounds. Further, using the same interpolation method, but this time on weekly cases subsampled from the daily cases in Bengaluru, we quantify the interpolation error on the Bengaluru case estimates (Fig. 7).
- (Correlation and lead times): We now have error bounds for correlation values and lead times and error bars for quadratic interpolation. We have provided 95% confidence intervals on the correlations (Table 5). The lead days using change-point analysis are now included with standard errors (Table 6) for a fixed threshold. In Table 7, we provide the mean and standard deviation of lead times (in days) for VCPD and VCPC at various STPs over a threshold range.

Thus we have addressed the impact of having 8-9 data points (which are more than the earlier 4-5) and have quantified the interpolation error and uncertainty in correlation.

False alarm rate and power are the parameters of interest for a fixed duration hypothesis testing problem. We have here a sequential hypothesis testing problem. False alarm rate is now replaced by the average run length to false alarm (ARL) under the null (under no infection or background infection level), in Section 'Change-point detection using cumulative sum (CUSUM)'. The analog of 'power' in this sequential setting is relative average detection delay (ADD) when there is indeed a change. Just as the power for various false alarm rates, obtained by varying the thresholds, gives the receiver operating

characteristics, Table 7 provides the relative ADD for various ARLs obtained by varying the threshold for decision.

We have added the above in the discussion section.

Comment 2

Another issue is the use of quadratic interpolation to estimate daily viral loads from the weekly samples. The reliability of this interpolation approach should be further validated, and uncertainties related to the interpolated values could be quantified.

Response: We have provided the following explanation in the discussion. We have increased our study window to a 77 day period giving us 8-9 data points per STP.

> We then used interpolation to derive daily samples from weekly wastewater data to determine correlation with the daily cases. We note that there is high correlation between the interpolated and actual values of viral load during during the Omicron wave (when there is a well defined peak along with a clear rise and fall in viral loads). However, we observe a dip in the correlation between actual and interpolated values during the baseline period with stable but low viral loads. This is also observed on viral load data from STPs in California. Over the three month period from Nov 2022 to Dec 2023, the method yielded correlations of around 20–30% but with much higher correlations during the Omicron wave (Fig. 6). As indicated earlier we have also addressed the impact of having 8-9 data points and have quantified the interpolation error and uncertainty in correlation obtained in Section 'Validating sample size and interpolation method'. We have quantified in the same section uncertainty in correlation due to error in interpolation (Table 5, last column).

Comment 3

The authors may consider a sensitivity analysis using the raw weekly data to evaluate the impact of interpolation on the results.

Response:

Thank you for the suggestion. We have analyzed the interpolation method now. We used daily viral load data from STP's in California and the daily cases data from BBMP. As indicated above we have provided error bars for the interpolated viral load estimates from a subsample of the data (Fig 6 and Fig 7).

Comment 4

Given the limited data, some conclusions drawn from the correlation analyses may need to be tempered. The authors should consider focusing conclusions on this specific case study and avoid broad generalizations. Claims could be qualified by emphasizing the uncertainties arising from data limitations.

Response:

Thank you. We have now rephrased the discussion to describe the limitations of the interpolation method, emphasized the uncertainties in correlation, and qualified the claims made.

Comment 5

The one-month study period may also constrain the generalizability of the findings. The authors could contextualize how focusing on this specific window within the Omicron wave may impact results compared to examining viral load and case correlations over longer timeframes. If feasible, expanding the analysis to cover multiple COVID waves could strengthen the study.

Response:

Our findings are limited to the Omicron wave primarily due to simultaneous availability of data on daily cases (due to comprehensive testing) and viral load data. In the discussion section we have also commented on the performance of the method during the wave period (when there is a well defined peak, clear rise in cases and fall).

We agree that covering multiple waves would strengthen the claims but post Omicron there was fall in reported cases likely due to lack of testing and decreased severity of disease in exposed/vaccinated populations. We believe the method would work in other situations when there is an outbreak and data (daily cases/viral load) is available.

Comment 6

Additionally, the manuscript could benefit from a discussion of potential confounding factors that may influence viral load and case correlations, such as changes in control measures and human mobility patterns over time. Comparing findings to studies of SARS-CoV-2 wastewater surveillance from other regions could provide useful context, even if a full comparative analysis is beyond the current scope.

Response: Thank you for the comment. We have discussed the confounding factors in the revision:

> During the study period, there were no mobility restrictions and other non-pharmaceutical interventions (such as wearing masks and quarantine measures) were recommended but not mandatory. We further assumed that there is a negligible floating population and sufficient homogenization. Under these assumptions an increase in viral load in an STP will correspond to an increase in case data in the corresponding catchment area. Our results are based only on this premise.

A unique feature of our work is the mapping of catchment area of each STP in Bengaluru. This helped correlate daily cases at the ward level with viral loads at respective STPs. We could not gather such granular data for daily cases in catchment areas along with corresponding STP viral loads in other regions. For example, cases at the catchments are not reported for California's STPs. Thus we were limited in providing a comparative analysis.

Comment 7

In summary, this study contributes to the growing body of research on wastewater surveillance for COVID-19. However, the conclusions that can be drawn are limited by the small number of data points. Revisions addressing and discussing the implications of these limitations could strengthen the robustness of the findings. I recommend that the authors consider the suggestions above to improve the manuscript. With appropriate revisions, this study could provide a valuable insight into the relationship between wastewater viral loads and COVID-19 cases during the Omicron wave in Bengaluru, while acknowledging the constraints posed by the available data.

Response: We thank the reviewer for their thoughtful comments. We hope our responses are satisfactory.

Authors’ Response to Reviewer 2

General Comments. The authors wrote about the localized wastewater surveillance and their application in early warning for SARS-CoV-2. The manuscript needs to have more discussion but overall, the model is interesting.

Response:

We have now expanded our discussion. We have mentioned the method’s limitations, quantified the uncertainty in correlation values, and discussed the unique features of our analysis.

Abstract

Comment 1

Please be aware that SARS-CoV-2 is the name of the virus. Covid 19 is the name of the disease. Check the whole document and fix if there are mistakes.

Response: Thank you for pointing out the difference. We have made changes at several places as appropriate. As an example, the first sentence of the abstract now reads: "Wastewater surveillance is an effective tool for monitoring the spread of infectious diseases like COVID-19."

Introduction

Comment 2

Line 3: poliovirus, not Poliovirus.

Line 4: antimicrobial resistance gene? Bacteria?

Line 5: Not all virus is an RNA virus.

Line 21: ... source tracking. What source tracking?

Line 31-32: Lead time of 2 days and as long as 1-2 weeks. Can we use this as a guidance? Check the result and see if the model showed at least 2 days lead with a good correlation with clinical cases.

Line 35: Please specify what virus. SARS-CoV-2?

Response: We have made the following changes.

- Typos on line 3 and line 4 are in the same sentence. The new sentence now reads:

  > Environmental surveillance of wastewater or sewage has proven highly effective for detecting diseases in populations, with past applications including the tracking of poliovirus, antibiotic-resistant bacteria, and antimicrobial resistance genes.

- Line 5: The word RNA has been dropped. The sentence now reads:

  > Wastewater surveillance entails continuous, systematic sampling and computing viral concentrations.

- Line 21: We have rephrased the sentence as follows:

  > Mapping catchment areas is crucial to localized wastewater surveillance, as it allows for the systematic identification of specific geographic regions contributing pathogens or contaminants to the wastewater system.

- Line 31-32: When computing correlations, we shift the case data relative to the viral load sequence by ±14 days, guided by the typical 1–2-week lead time observed in disease dynamics. A lead of two days also gives a high correlation. However, a slightly better correlation is attained when there is no lead. In the revised manuscript, have a fixed window size of length 77. We observe that a 3 day shift on an average gives the maximum correlation.
- Line 35: We have specified the virus. The sentence is changed to:

> The SARS-CoV-2 virus is shed in respiratory secretions as well as feces.

Method

Comment 3

Line 65-66: Please specify why grab method was chosen, what are their limitation, around what time those samples were collected?
How did the author measure the RNA copies of SARS-CoV-2? qPCR? What are the primers and probes, or kit used? What PCR machine was used? What is the limit of detection? What is the positive control used?

Response: Thank you for your pertinent questions. As mentioned in the manuscript, our viral load data comes from Lamba et. al. (Reference numbered 13 in the revised manuscript) While we have not collected or tested the samples ourselves, some of the information requested can be found in the 'Methods' section of their paper. We now mention it here for completeness sake.

- STPs in Bengaluru do not have autosamplers, hence it was not possible to obtain composite samples. Grab samples are known to have slightly higher variability than composite samples.

- Samples were collected during 0800–1400 hours.
- Name of the kit: GenepPath Dx CoViDx One v2.1.1TK RT-qPCR kit.
- Since the kit provides only qualitative test, an online viral load calculator was used to quantify the viral load: https://coviquant.genepathdx.com/.
- The limit of detection is 2-3 copies of SARS-CoV-2 RNA genome equivalents per reaction.
- Positive control: RNA containing the N, RdRp, and E-genes.

Comment 4

Line 105-121: Did the author try to normalize the data by using flow and population? Please try and see if it works better with the model.

Response: We did consider normalizing by the flow. The flow data is available at 5-minute intervals on the BWSSB website. However, we found discrepancies in the data that we could not resolve. For example, the inlet flow during December 2024 has a single value of 35.699 MLD for Agaram STP (35 MLD). Source: https://stpp.bwssb.gov.in/stp.aspx?id=1.

Secondly, the viral load is expressed as RNA copies/ml. This quantity is proportional to the fraction of the population infected in the catchment area, and therefore no normalisation with respect to population is needed. We ensured that the catchment area was accurately mapped to the respective STP.

Comment 5

What is quadratic interpolation and how did the author did it? What did author mean by clip any negative result? Cutting the data or use 0? Why don't use a limit of detection number?

Response: We clarify below.

- Quadratic interpolation is a common interpolation method that takes three points and fits a smooth curve (a parabola) passing through them. We use this method to estimate the daily viral loads from weekly viral loads. Daily viral loads are estimated by taking three consecutive weekly sample values at a time. We use the `interpolate` function from the Pandas library in our implementation. Reference: https://pandas.pydata.org/docs/reference/api/pandas.DataFrame.interpolate.html
- By clipping, we mean the following. If the interpolation method estimates a negative value for the viral load on a day, we consider the viral load to be zero on that day. This happens when the weekly viral loads are close to zero during the baseline period.
- We understand the limit of detection to be a property of the testing kit. We are uncertain about its potential application in estimation.

Quadratic interpolation is now better explained in the manuscript.

Comment 6

Line 117-121: How did the author combine 2 STP and replace their daily viral loads with weighted average?? Please describe 1 calculation as example.

Response: To combine the viral loads of two STPs, we calculated a weighted average based on their treatment capacities. We have included the following example calculation in the revised manuscript.

Suppose STP A has a treatment capacity of 50 million liters per day (MLD) and a daily viral load of 2.5×10^3 copies/ml, while STP B has a treatment capacity of 100 MLD and a daily viral load of 3.5×10^3 copies/ml.

The weighted average viral load is calculated using the formula:

$$\text{Weighted Viral Load} = \frac{(V_A \cdot C_A) + (V_B \cdot C_B)}{C_A + C_B}$$

where:

- V_A and V_B are the viral loads of STPs A and B in copies/ml,
- C_A and C_B are the treatment capacities of STPs A and B in MLD.

Substituting the given values:

$$\begin{aligned}\text{Weighted Viral Load} &= \frac{(2.5 \times 10^3 \cdot 50) + (3.5 \times 10^3 \cdot 100)}{50 + 100} \\ &= \frac{(1.25 \times 10^5) + (3.5 \times 10^5)}{150} \\ &= \frac{4.75 \times 10^5}{150} = 3.17 \times 10^3 \text{ copies/ml.}\end{aligned}$$

Comment 7

For clinical cases. Was it the data based on hospital report? Or based on patient residency?

Response: Thank you for the question. We have added the clarification.

A case is attributed to the patient's residence.

Comment 8

Line 148-159: CUSUM. Please mention the full words before abbreviating it.

So what was the h-value that applied here?

How to measure the average run length? Expected time until a change was detected? Is it the same as what mentioned at line 157?

Response: We have made the following corrections.

- The title of the section is changed to **Change-point detection using cumulative sum (CUSUM)**.
- The threshold value for h is 20. This value is mentioned in footnote (d) of Table 5 (Table 6 in the new version). We chose it by trial-and-error. The value is set high enough to have almost zero chance of false positives for most STPs. This allows us to compare the lead time between cases and viral loads. In the revised manuscript, a new table (Table 7) is added to show each STP's mean and standard deviation of the lead time.
- There is no closed formula for computing average run length (ARL) as a function of the pre-and-post-change distribution parameters. Analytically approximate formulas are given in [1]. However, we did not use them.
- Line 157 refers to the change point time τ, which is different from ARL.

[1] W. D. Ewan and K. W. Kemp, "Sampling inspection of continuous processes with no autocorrelation between successive results," *Biometrika*, vol. 47, no. 3-4, pp. 363–380, Dec. 1960.

Results

Comment 9

Table 1 is too long. Maybe can be mentioned as supplementary information. Please make a summary of table 1. Like how many wards and their range of MLD.

Response:

Thank you for the suggestion. We debated whether to include this table (now Table 4) in the main text or supplementary material. Ultimately, we felt it should be part of the main narrative, as it addresses a critical data gap for localized analysis and has broader implications for the surveillance of other pathogens. We hope we can retain it in the main text.

Comment 10

Line 182: Correlation analysis was not mentioned in the methodology section. Line 184: This should be in method section

Response: We agree. We have moved the lines 183–185 to the methodology section.

Comment 11

Why was Pearson analysis considered?

Response: We opted for Pearson correlation because preliminary analysis indicated a linear relationship between viral loads in wastewater and reported COVID-19 cases within the catchment areas. This linear association made Pearson correlation an appropriate measure to quantify the strength of this relationship.

Preliminary analysis is added to the section on 'Computing Correlations'.

Comment 12

Table 2 and 3: How the author concluded that there is no lead? there are lead number with good correlation there.

Response: We found different lead times for different window lengths in the original approach. Since the lead times were inconsistent, we could not conclude if there was a lead. In hindsight, we realized that the inconsistency was due to the small number of samples in each window. We have revised our approach and now have a fixed window length of 77. We also have a new section that validates the sample size. Table 2 and Table 3 are not present in the revised version. In the revised approach, we notice a lead of three days with the interpolated daily viral loads. We interpret the lead as follows:

> (*Results section*) At first glance, it suggests that viral load leads the cases by three days. The observed three-day lead in the interpolated daily viral load data is an artifact of the interpolation process, rather than a true reflection of the relationship between viral loads and case numbers. Since viral load measurements are taken weekly, interpolating this data to a daily frequency introduces a lead that may not accurately represent the actual timing between viral loads and cases. Therefore, this lead should not be considered significant. A lead of seven or more days, however, would be more meaningful, as it aligns with the weekly sampling schedule and could provide useful information for early warning.

Comment 13

Figure 5: How was the epi curve data obtained. It should be mentioned at the method section.

Response: The epi curve data shows the citywide reported cases. We have added the following sentence to the methodology section to clarify the source of data:

Citywide cases are calculated as the sum of cases reported across all wards.

Comment 14

How to determine the case shift?

Response: In the revised manuscript, the method for calculating the shift differs from that used in the original manuscript. In particular, we have only one window of length 77 for cases and viral loads. The relevant portion of the revised text is replicated below:

- **Time period** T: Let T denote the dates from 15 November 2021 to 31 January 2022. There are $n = 77$ days in this period.
- **Shift parameter** η**:** The parameter η is an integer between -14 and 14 that indicates a shift in the time period T. T_η refers to the 77-day period starting from η days before 15 November 2021. For example, T_3 denotes the dates from 12 November 2021 to 28 January 2022. Similarly, T_{-2} denotes the dates from 17 November 2021 to 2 February 2022.
- **Case Vector** ($\mathbf{c} = (c(1), \ldots, c(n))$): The vector representing the daily COVID-19 cases reported within the catchment area of S during the period T.
- **Interpolated Daily Viral Load Vector** ($\tilde{\mathbf{v}}_\eta$): The vector obtained by interpolating viral load measurements taken weekly from S using quadratic interpolation. The viral loads are for the days in the period T_η.

When calculating the correlation between cases and viral loads, we keep the case vector fixed i.e., the case vector is always for the period T. The viral load vector is shifted relative to the case vector for $\eta \in \{-14, \ldots, 14\}$. We are interested in

the maximum correlation over all shifts. The maximum correlation $\rho_{\mathbf{c}\tilde{\mathbf{v}}}$ and the corresponding shift $\eta^\star$ are defined as:

$$\rho_{\mathbf{c}\tilde{\mathbf{v}}} := \max_{\eta \in \{-14,\ldots,14\}} \rho_{\mathbf{c}\tilde{\mathbf{v}}_\eta} \tag{1}$$

$$\eta^\star := \operatorname*{argmax}_{\eta \in \{-14,\ldots,14\}} \rho_{\mathbf{c}\tilde{\mathbf{v}}_\eta} \tag{2}$$

Comment 15

Figure 6: This should be discussed in the discussion section. How did the data compared based on windows of size?

Response: In the revised manuscript, we have only one window size. Hence, figures 5-6 and the related discussion are removed.

Comment 16

Line 198-219: Need to be in method section. Why did the author explain analysis method at the result section?

Response: We agree. We have moved the lines 198–219 to the methodology section.

Comment 17

Just curious, if the viral loads were measured weekly, is it possible to use the weekly average clinical cases for CUSUM analysis? If yes, why it was not conducted? Is it because the window is too small and it will be hard to get correlation?

Response:

Thanks for the comment. We were exploring the possibility of a finer granularity in days for the lead time. If the correlation was done on weekly data, we would anyway have to interpolate to the finer granularity of days. Hence we followed our approach.

Comment 18

Based on Figure 9, I can see there was early warning where the peak was detected after the VCPD.

Response: Early warning does not imply a peak-to-peak delay between viral loads and the cases. We have also addressed this in Comment 19.

Comment 19

Line 234-243: Early warning is not well defined. If the peak from wastewater followed by peak from clinical cases after several days, does not it count as early warning? Based on Fig 9 I can see that. What is the threshold for early warning? Figure 8 says 4 days. Please discuss from figure 9, what was the range for VDPC for all the STPs, did it consider as early warning. If not, why?

Response: Thank you for your comment. The lack of definitions has led to confusion. We have corrected this.

- We agree that the terms early warning, Lead-VCPD, and Lead-VCPC were not defined. We have now added these definitions just before the results section.
- The threshold for early warning is 1 day, i.e., if the value of Lead-VCPC > 0, we consider it an early warning.

- In Fig. 8, the value of Lead-VCPC = 4 days.
- The VCPC in Fig. 9 ranges from 2021-12-24 to 2022-01-17. The range cannot be seen in the figure itself but we must refer to the leadtable (Table 7 in the revised document). We have edited the caption to Fig. 9 to make it more clear:

  > The alarm dates for each STP are indicated with dotted lines (from Table 7). The blue and the green dotted lines indicate Cases-CP and Lead-VCPC, respectively.

  As per our definition, early warning occurs for the first five STPs of Table 7. In Fig. 9, the green dotted line appears before the blue dotted line for these sites.

Discussion

Comment 20

In my opinion, the discussion part is still needing more works.

Line 228-231: Just repetition from method.

Response: As mentioned earlier in response to the general comments, we have expanded the discussion section.

- Line 228-231: We have removed the repeated lines.

Comment 21

Author only mentioned there is a good correlation between the wave of wastewater and clinical but no further discussion. Why are there good correlation? Is it the model? If yes how about comparing the model used here with another model? Please find more literature to discuss. For example, please compare to ARIMA model.

Response:

Our hypothesis was that viral loads have a good correlation with daily cases and therefore could be used as a proxy when testing is not comprehensive. This is because people with infection shed the virus into the waste water. Our methods were chosen to justify this hypothesis and quantify this correlation.

We were primarily interested in testing if the two sequences, namely viral load and daily cases, were correlated. Hence we did not feel the need to use a parametric model like ARIMA.

Comment 22

An increase in wastewater viral load does not necessarily an outbreak indicator.

Response:

We agree. We have modified the above observation in the last concluding paragraph of the discussion section.
