# Supplementary material for: Localized wastewater surveillance showed correlation but no early warning during Bengaluru’s Omicron wave
Source: PLOS Glob Public Health. 2026 Apr 10;6(4):e0004684. doi: 10.1371/journal.pgph.0004684 (PMC13068238; doi:10.1371/journal.pgph.0004684)
Supplement: Attachment — Submitted filename: PLOS_Rebuttal_auresp_2.pdf [file pgph.0004684.s014.pdf]

Responses to Reviewers' Comments for Manuscript PGPH-D-24-02064R1

# Localized wastewater surveillance showed correlation but no early warning during Bengaluru's Omicron wave

Addressed Comments for Publication to

PLOS Global Public Health

by

Siva Athreya, Farah Ishtiaq, Tarun Khandelwal, Jagadish Midthala, Chitra Pattabiraman, Lakshminarayana Rao, Rajesh Sundaresan and Reshma Mohan Thattaramppilly

Dear Dr. Amarasiri,

Please find enclosed the revised version of our previous submission titled " Localized wastewater surveillance showed correlation but no early warning during Bengaluru's Omicron wave" with revised manuscript number PGPH-D-24-02064R1. We want to thank you and the reviewers for the valuable comments. In the second revision, we have addressed the queries raised during the review process. A summary of the main modifications and point-by-point responses to the comments are given below.

Sincerely,

Jagadish Midthala

**Note:** To enhance the legibility of this response letter, the editor's and reviewers' comments are typeset in boxes. Changes to the manuscript are given in boxes with gray background. Line numbers refer to the line numbers in the original manuscript. In the marked manuscript major changes are marked in blue color.

## Authors' Response to the Editor

**General Comments.** One reviewer has highlighted the necessity to improve the methodology section.

**Response:**

We thank the reviewers for their careful reading of the manuscript and appreciate the feedback given. We have revised our manuscript based on the second reviewer's comments. The main changes are listed below.

1. We have added more details to the methodology section.
2. We have highlighted 4-5 limitations of the method in the discussion.
3. A few figures in the main text are moved to the supporting information.

## Authors' Response to Reviewer 1

**General Comments.** I recommend this manuscript for publication in its current form. The authors have thoroughly addressed all major methodological concerns through expanded analysis, validation, and appropriate uncertainty quantification. The conclusions are well-supported and properly qualified, making a valuable contribution to understanding localized wastewater surveillance during the Omicron wave.

**Response:** We thank the reviewer for their positive feedback and are glad that they found the manuscript suitable for publication in its current form.

## Authors' Response to Reviewer 2

**General Comments.** The author has submitted a revised version of the manuscript in response to previous feedback. The revised manuscript is better written and demonstrates improved clarity. I have some comments remaining for further revision.

**Response:** Thank you for the kind feedback. We have addressed the remaining comments as detailed below.

### Abstract

**Comment 1**

Please include the correlation value(s) referenced.

**Response:** Since we do not give point estimates for correlation values, we have specified the typical confidence interval. We have added the following sentence.

> A typical confidence interval for the Pearson correlation between clinical cases and wastewater viral loads at an STP is approximately 0.56–1.00, based on the median bounds across the STPs.

**Comment 2**

Kindly remove the statement mentioning who conducted the surveillance in 2021, as it is not relevant here.

**Response:** The sentence now reads as follows:

In August 2021, a citywide surveillance effort was initiated in Bengaluru to analyze viral loads from 28 sewage treatment plants (STPs).

**Comment 3**

Please clarify what is meant by “correspond” in the context used.

**Response:** We have reworded the sentence and mentioned the correlation estimates to address the earlier comment (Comment 1).

## Author summary

**Comment 4**

The first bullet point is not a result of this study and should therefore be removed.

**Response:** We have removed the point.

## Introduction

**Comment 5**

The explanation of viral load currently in line 46 should be moved earlier, to line 41, for better context.

**Response:** Thank you for the comment. Viral load is now defined before its usage.

**Comment 6**

Please clearly state the aims of the study in the final paragraph of the introduction. Refer to line 63 of the methodology for guidance.

**Response:** Thank you for the comment. The last paragraph now reads:

Our main objective in this study is to quantify the correlation between observed wastewater viral loads at STPs and reported cases in Bengaluru, a city in India with a population of 14 million. We chose the period around the first Omicron wave, i.e., from 15 November 2021 to 31 January 2022. During this period the viral load data in wastewater samples from 26 sewage treatment plants (STPs) were available from [13] and due to comprehensive testing policy, daily cases data were available at https://site.bbmp.gov.in/indexenglish.html. We first map the catchment areas from Bengaluru for each of these STPs. We then perform correlation analysis and implement a refined change-point detection analysis to evaluate if viral load leads cases in the city during the Omicron wave.

## Method

**Comment 7**

As noted for the abstract, please remove the sentence on line 69 referencing who conducted the surveillance.

**Response:** We have dropped the reference.

**Comment 8**

Line 69: What was the normalization method used? Please specify.

**Response:** The values were normalized to z-scores by subtracting the mean and dividing by the standard deviation. However, we have removed this reference because it is a secondary source and the website has not been updated.

**Comment 9**

Line 73: The researcher's name mentioned here should instead be included in the Acknowledgments section.

**Response:** We have included the researcher as a co-author in the revised manuscript.

**Comment 10**

Figures 3 and 4 could be moved to the supplementary material. Figures 1 and 2 are sufficient for illustrating the map.

**Response:** Figures 3 and 4 are now supporting files S1 Fig. and S2 Fig., respectively.

**Comment 11**

Lines 128–132: Please include only the formula; the example calculation can be moved to the supplementary section.

**Response:** We have moved the example to the supporting information.

**Comment 12**

When describing the correlation analysis, please explain the assumption of linearity between viral load and COVID-19 case numbers. Why is a linear relationship appropriate here?

**Response:** In our analysis, the relationship between viral loads and reported cases turns out to be approximately linear, as evidenced by visual inspection of the data (S5 Fig.) and post-hoc correlation analysis (Table 2); this linearity is established only retrospectively, once both datasets are available.

We have added references at the beginning of the section on computing correlations. Previous studies have reported linear and nonlinear associations between SARS-CoV-2 RNA concentrations in wastewater and COVID-19 case counts in catchment areas. For instance, linear correlations have been observed in monitoring efforts in Scotland. To quote from Fitzgerald et. al.:

> "Daily WWTP SARS-CoV-2 influent viral RNA load, calculated using daily influent flow rates, had the strongest correlation ($\rho > 0.9$) with COVID-19 cases within a catchment. As the incidence of COVID-19 cases within a community increased, a linear relationship emerged between cases and influent viral RNA load."

From Rabe et. al. whose work on California STPs revealed both linear and polynomial relationships between cases and viral loads.

> "There was also visual evidence that the relationships between wastewater concentrations and case incidence rates were not linear for every sewershed and variant period (Figure 3). We tested for evidence of nonlinear relationships (comparing linear, quadratic, and cubic models) between SARS-CoV-2 concentrations and corresponding sewershed case incidence rates during all variant periods. This analysis suggested that a linear model provided the best fit for only five of the sewershed-laboratory-period combinations. For all other subset datasets, quadratic or cubic models improved model fit (Table 4). "

**Comment 13**

Figure 5 should be moved to the supplementary material. Results should not be presented in the Methodology section.

**Response:** Moved to the supporting information section.

**Comment 14**

The explanation of the interpolation method should be made more concise. The associated calculation results (Figures 6 and 7, Tables 1 and 2) should be moved to the supplementary section, as these are results.

**Response:** We have moved the figures and the tables to the supporting information section. We now explain the interpolation method as follows:

> We use quadratic interpolation to estimate missing values in our data. We implement this using the interpolate() function from the Pandas Python module. If the interpolation outputs any negative values for viral loads, we set them to zero.

**Comment 15**

The same applies to the CUSUM analysis: Figures 8 and Table 3 should be relocated to the supplementary material.

**Response:** We have moved the figure and the table to the supporting information section

**Comment 16**

For Table 5, please define what "PCv" refers to.

**Response:** The correlation values $\rho_{\mathbf{cv}}$ and $\rho_{\mathbf{c}\tilde{\mathbf{v}}}$ were defined in the section on the validating sample size. We have now defined it in the table-footnote also.

### Results

**Comment 17**

The interpolation method used for a small dataset is interesting. Please discuss the interpretation of the results presented in Figures 6 and 7. What does the error factor represent? What values are considered good or poor in this context?

**Response:** Thank you for the comment.

We have now added the following explanation:

> The error factor is the ratio of the estimated (interpolated) value and the true viral load. . . The y-axis shows the error factor. Each point represents the mean error factor for a given week, and the vertical whiskers show the standard deviation, indicating the variability of the error factor across all points within that week.

We also state the role of the error bars explicitly:

> The weekly error bars in S4 Fig and S5 Fig. are not directly used in the calculation. However, based on the error bars shown in the figures, we assume that the interpolation method introduces a 25 % standard deviation error in each viral load value. This assumption is used in Step 2 below.

## Comment 18

How small can the dataset be for this interpolation method to still be effective?

**Response:** One measure of effectiveness is the width of the confidence interval for correlation. There is no sharp cutoff for how small the dataset can be. At the current (weekly) frequency we see that the median confidence interval is 0.56-1. If we increase the time period to 14 days, then the error bars become bigger (as shown below). We found that the lower bound of the intervals become close to 0.1 for a few STPs at this point. So, the median confidence interval slowly degrades from 0.56-1 to 0.1-1 as we go from 7 to 14 day frequency.

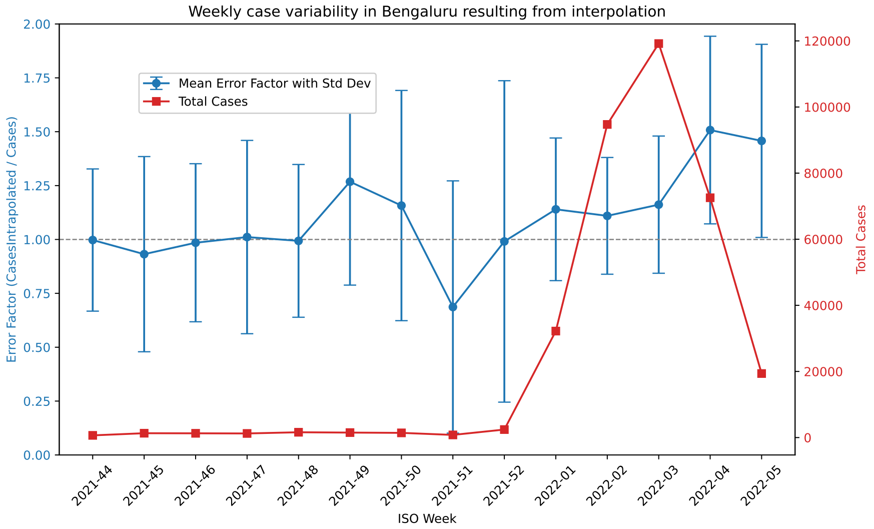


**Remark.** We make the following change to the script `code/blr_cases_error_bar.py` to get the above plot.

```
#df['CasesIntrapolated'] = df['Cases'].where(df['SampleDate'].notna())
df['CasesIntrapolated'] = df['Cases'].where(df['Date'].dt.day%14 == 1)
```

**Comment 19**

Please include a discussion of the study's limitations and suggestions for improvement in future work.

**Response:** We have added the limitations of the work and possible improvements in the discussion:

Coverage of STPs in Bengaluru is extensive, capturing wastewater from a large proportion of the population. However, some portion of wastewater remains untreated and is discharged into open drains. The study by [53] examined viral loads in such open drains, providing complementary insights.

Large sewage treatment plants (STPs), like K&C Valley, cover a wide area and mix wastewater from many neighborhoods, making it harder to detect localized outbreaks. This reduces the effectiveness of wastewater surveillance for specific areas. To improve this, we suggest sampling from sentinel manholes at key neighborhoods or high-density areas. Sampling from these manholes would allow for better detection of local viral trends.

We compute the citywide viral load by weighted average of viral loads of STPs, weighted by capacities of STPs. A more accurate method to compute the weighted viral load would be to use daily sewage flow data instead of capacities. However, reliable flow data from BWSSB is not available during the Omicron period.

Our manual mapping of wards to STPs, based on inspection of the sewerage network and expert consultation, does not easily scale to other cities. It may be possible to automatically map regions to STPs using the sewerage network and the city's topography. Given a detailed network file, such as a GIS file, and elevation data, flow directions can be traced by following pipe connections and slopes, since wastewater generally flows downhill. This would allow automated assignment of areas, such as wards, to their respective STPs. While practical challenges like missing data, pumping stations, or undocumented connections may affect accuracy,

in principle, accurate and updated network and topographic data would enable systematic and reliable mapping. This approach can be explored as future work to improve the precision and scalability of wastewater surveillance efforts.

**Comment 20**

Line 393: Is the observation discussed here possibly due to limitations in the sampling strategy or data availability? Would more complete data potentially lead to different outcomes?

**Response:** We have mentioned one limitation of sampling from inlets of large STPs (response to Comment 19). If more complete data were available, we would get sharper estimates for correlation values. This would help in a few STPs like Bellandur, Jakkur and Yelahanka which have wide CIs of 0.34-1, 0.33-1 and 0.0-0.88, respectively. But our main conclusions about correlation and lack of lead times would still hold for most STPs.
