# Supplementary material for: Localized wastewater surveillance showed correlation but no early warning during Bengaluru’s Omicron wave
Source: PLOS Glob Public Health. 2026 Apr 10;6(4):e0004684. doi: 10.1371/journal.pgph.0004684 (PMC13068238; doi:10.1371/journal.pgph.0004684)
Supplement: Attachment — Submitted filename: response-to-review.pdf [file pgph.0004684.s015.pdf]

Response to Authors' Self Review

Previous review status: The manuscript was accepted for publication at PLOS Global Public Health. But we requested that the publication be put on hold and the manuscript be sent back to us, because we wished to investigate a discrepancy between (a) the data used in our analysis and (b) the supplementary data in a paper that appeared later.

Discrepancy analysis: The later paper has now been added as reference [13] in the revised manuscript:

[13] Daniel RF, Kannan SK, Daroch N, Ganesan S, Mozaffer F, Srikantaiah V, et al. Identifying bellwether sewershed sites for sustainable disease surveillance in Bengaluru, India: a longitudinal study. The Lancet Regional Health-Southeast Asia. 2025;39:100619. doi:10.1016/j.lansea.2025.100619.

We have since successfully resolved the discrepancy as follows.

The discrepancy was due to an inadvertent data-conversion error in [13, Supplementary Table S4]. As confirmed by the corresponding author of [13], the correct values of the raw viral loads (copies/ml) for December 2021 are 1.25x those reported in [13, Supplementary Table S4, column G]. The data that we had used in our analysis was shared independently of [13], and had the correct values for the viral copies/ml. Hence, there is no change to our analysis, derived data, and conclusions.

Updates to the manuscript: The only changes to the revised manuscript are:

1. Addition of the reference [13] as indicated above;
2. A note in S4_Table.pdf pointing out the inadvertent error in [13], and that we have used the correct values in our analysis.
3. Change in author list: One of the coauthors of an earlier version, Dr Jagadish Midthala, decided to withdraw from authorship. The revised manuscript does not include him as a coauthor, but gratefully acknowledges his contribution to the manuscript.
