# Supplementary material for: Localized wastewater surveillance showed correlation but no early warning during Bengaluru’s Omicron wave
Source: PLOS Glob Public Health. 2026 Apr 10;6(4):e0004684. doi: 10.1371/journal.pgph.0004684 (PMC13068238; doi:10.1371/journal.pgph.0004684)
Supplement: Attachment — Submitted filename: PLOS_Wastewater_Manuscript_Response.pdf [file pgph.0004684.s016.pdf]

Response to Reviewers:

Reviewer #1:The authors have fully and transparently resolved the data discrepancy and other issues raised, without affecting the study's conclusions. I recommend acceptance of the manuscript.
Response: We thank the reviewer for the feedback and the recommendation.

Reviewer #2: This manuscript addresses a timely and important topic in covid19 surveillance by examining the relationship between wastewater viral loads and clinical case trends across multiple sites. As wastewater-based epidemiology continues to evolve as a complementary tool for infectious disease monitoring, especially for enteric and respiratory viruses, studies that evaluate its accuracy, temporal dynamics, and analytic approaches are essential. The authors present relevant data with clear public health implications; however, several methodological clarifications, expanded discussion points, and additional statistical context are required to enhance the rigor, interpretability, and impact of the findings. The comments below are intended to strengthen the manuscript and support its suitability for publication in PLOS Pathogens.
Comment 1: Abstract

Please include the confidence-interval values for city-level clinical data versus aggregated viral load so that the similarity with localized viral load trends can be justified.
Response: We have added a line that provides the city-level confidence interval.

Comment 2: Introduction

Line 4: Please revise this sentence. Antimicrobial resistance (AMR) and antimicrobial resistance genes (ARG) surveillance represents a recent application of wastewater surveillance. It should not be presented as a previous or early use of environmental surveillance. The cited reference is correct; however, the interpretation requires adjustment.
Response: We have addressed the comment by modifying the sentence. We now say 'and emerging applications such as detecting antimicrobial resistance' so that it is not misinterpreted as an early use.

Comment 3: Line 29: Please define the criteria used to classify a "strong correlation."
Response: We have removed the word "strong". When we revisited the references, we felt that there was correlation but the word strong may be misleading.

Comment 4: Line 33: Please elaborate on the reasons why increased sampling frequency strengthens correlation analysis.
Response: We have addressed the comment. The reason is a reduction in estimation errors.

Comment 5: Line 34: Please provide a clear definition of "lead times."
Response: We have added the definition of lead time. (It is the number of days by which the wastewater viral load trajectories lead the clinical case trajectories.)

Comment 5: Methods
Lines 118–119: Please clarify the Limit of Detection (LoD) and Limit of Quantification (LoQ). If the LoD values are available, please explain why they were not used in place of zero. In addition, please clarify whether applying the LoD threshold would alter the results of the correlation analysis.

Response: We have addressed the comment. We have added a line explaining the methodology used for replacing interpolation values which are negative, projection to the nearest value on the set of valid viral loads, which is a mapping to 0. Since 0 is the nearest point, limit of detection or quantification does not enter the calculation.

Comment 5: Lines 122–124: Please explain why the sewage treatment plant (STP) capacity was used rather than daily or weekly flow-rate averages.

Response: We have added an explanation. The main reason is that the STPs are operating at capacity.

Comment 5: Figure 4: Please include the units for viral load. In addition, the final portion of the figure appears to show decreasing clinical cases while viral loads increase. This pattern appears inconsistent with other STPs shown in Figure 5. Please discuss any potential explanations.
Discussion
Response: We have indicated the unit as copies/ml. We have now placed another STP graph for illustration. We don't have any potential biological explanation for Hebbal STP waste water viral load rising, and had just reported the observed value. But since this was perhaps misleading, we have replaced it with another STP graph (composite of Rajacanal and Horamavu STPs).

Comment 6: Lines 343–349: I recommend expanding this section by introducing standard correlation strength categories (for example, low, moderate, and high) and discussing the rationale behind these classifications. Please

also describe the statistical methods used, why they are appropriate, and whether they have been commonly applied in similar studies. Highlight which STP demonstrated the highest and lowest correlations and consider providing potential explanations, such as geographic context or differences in data availability.
Response: We have expanded the discussion. The threshold for low is 0.50, the threshold for moderate is 0.80, and the rest is high. These thresholds are based on basic clustering on the data (0.49, 0.79, 0.79, 0.80, 0.82, 0.84, 0.87, 0.88, ...). On statistical methods, the correlation method is classical text book material and the discussion now reflects the same. We have also highlighted the ones with low and moderate correlation and attributed the reasons to abnormally low or missing viral loads.

Comment 7: Lines 355–357: Please clarify the reason for the low viral loads observed during this period. Review lines 420–422 and assess whether the reported error bounds are sufficiently narrow to support confidence in the findings.
Response: Thank you for the comment. The low values are during the baseline period due to the delay in arrival of the Omicron wave and also the testing policy that was followed during that time. We hope the phrase baseline period helps clarify.

Comment 8: Lines 360–362: Please justify the interpolation method used. A brief explanation regarding why interpolation is appropriate for this dataset would strengthen this section.
Response: We have addressed this comment and added a line. The interpolation is needed as the data is available once in seven days and we use use a quadratic-interpolation to follow the rising or falling trends closely without introducing a lead bias.

Comment 9: Lines 363–372: This portion reads more like a methods description rather than a discussion. Please revise to address the advantages of the modelling approach, cite comparable studies that have applied similar models, and discuss alternative models that could have been used, including relative strengths and limitations.
Response: We have now highlighted the modelling advantage of looking at differences which enables us to use the CUSUM. However, the limitation is that we have to now work with fewer samples, which is also indicated. In addition, we also highlight the advantage of CUSUM itself which is a sequential technique that accumulates evidence to give lower detection delay at given false alarm rates.
